# Iatrogenic Pectus Deformities after Sternotomy in Patients with Down Syndrome

**DOI:** 10.1055/s-0045-1809415

**Published:** 2025-07-10

**Authors:** Davi de Podestá Haje, Jorge Henrique Carlos Aires, Talita Virginia Pinto de Sousa, Fernando Aurélio de Sá Aquino

**Affiliations:** 1 Centro Clínico Orthopectus, Brasília, DF, Brazil; 2 Department of Orthopedic Surgery, Hospital de Base do Distrito Federal, Brasília, DF, Brazil

**Keywords:** deformity, Down syndrome, iatrogenic disease, pectus carinatum, pectus excavatum, sternotomy, deformidades, doença iatrogênica, esternotomia, pectus carinatum, pectus excavatum, síndrome de Down

## Abstract

**Objective:**

To evaluate the incidence of pectus deformity after sternotomy in patients with Down syndrome, in addition to the clinical and radiographic characteristics.

**Methods:**

There were 20 patients with sternotomy history during childhood and a control group ( *n* = 20). The chest was clinically evaluated for the presence and type of pectus deformity, severity, and the clinical sternal body length. Radiographic examinations were used to evaluate any abnormalities.

**Results:**

From the total, 85% ( *n* = 17) presented with pectus deformities (41% lateral pectus carinatum, 65% mild severity, and 29% little flexibility). In the control group, deformity occurred in 5% ( *n* = 1). In the sternotomy group, 40% ( *n* = 8) had a clinically shortened sternum, which did not occur in the control group ( *p* = 0.01). Radiographic examination of the sternotomy with pectus group showed posterior angulations in the manubrium (10%), sternal shortening (38%), and irregularities in the sternal body (70%); furthermore, 36% of the children had all sternal growth plates closed, and 10% had early sterno-manubrial fusion, which did not occur in the control group.

**Conclusion:**

Patients presented a high incidence of pectus deformity after sternotomy (mostly mild and of the lateral carinatum type), with radiographic changes suggestive of abnormal sternal growth.

## Resumo

**Objetivo:**

Avaliar a incidência de deformidade *pectus* após esternotomia em pacientes com síndrome de Down, além das características clínicas e radiográficas.

**Métodos:**

Foram estudados 20 pacientes com histórico de esternotomia durante a infância e um grupo controle ( *n* = 20). O tórax foi avaliado clinicamente quanto à presença e tipo de deformidade *pectus* , gravidade e comprimento clínico do corpo esternal. Exames radiográficos foram utilizados para avaliar quaisquer anormalidades.

**Resultados:**

Do total, 85% ( *n* = 17) apresentaram deformidades *pectus* (41% *carinatum* lateral, 65% gravidade leve e 29% pouca flexibilidade). No grupo controle, a deformidade ocorreu em 5% ( *n* = 1). No grupo de esternotomia, 40% ( *n* = 8) apresentaram esterno clinicamente encurtado, o que não ocorreu no grupo controle ( *p* = 0,01). O exame radiográfico do grupo com *pectus* mostrou angulações posteriores no manúbrio (10%), encurtamento esternal (38%) e irregularidades no corpo esternal (70%); além disso, 36% das crianças tinham todas as placas de crescimento esternal fechadas, e 10% apresentaram fusão esterno-manubrial precoce, o que não ocorreu no grupo controle.

**Conclusão:**

Os pacientes apresentaram uma alta incidência de deformidade *pectus* após esternotomia (principalmente leve e do tipo *carinatum* lateral), com alterações radiográficas sugestivas de crescimento esternal anormal.

## Introduction

Pectus deformities are idiopathic in most cases, but may also be iatrogenic, 1 2 congenital, or pathological (such as, Marfan syndrome). 3 It is mostly noticed at around 10-years-old, often worsening during puberty. 4

Sternal growth occurs through endochondral ossification 5 6 and presents as growth plates between its osseous segments and the costochondral junctions, which was originally described by Haje and Bowen. 4 The onset of sternal growth disorders during the prenatal and developmental periods can result in a disproportion between the sternum and the ribs.

Haje et al. 7 described that lesions in the sternal growth plates of rats can generate pectus deformities. Primary lesions or anatomical disarrangements of the sternal growth plates resulting from sternotomy during cardiac surgery seemed to have caused deformities during children's growth period. 2 8 9 Another possible cause would be the persistence of a macroscopic gap between the growth plates or the two halves of the sternal body after closing the sternotomy. 9

Lateral radiographic views revealed early closure of sternal growth plates in patients with pectus, especially in the superior carinatum or Currarino type, resulting in shortened and curved sternal bodies. 10

The first reported case of iatrogenic pectus deformity in the literature was in 1995, involving pectus carinatum after sternotomy for the treatment of cardiac malformation, which was satisfactorily treated with the Dynamic Chest Compressor 1 (DCC 1) brace, 8 first described by Haje and Raymundo in 1979. 11 Another case of iatrogenic pectus carinatum has been described. 12 However, the number of cases is possibly underreported, especially those with mild deformity, which may not be prioritized by attending physicians or family members.

Haje et al. 9 have also classified idiopathic pectus deformities in terms of clinical type, severity, and flexibility; 1 4 9 but these factors have not been analyzed in the setting of iatrogenic pectus deformities.

The objective of this study was to evaluate the prevalence of iatrogenic pectus deformity in skeletally immature patients with Down syndrome who underwent sternotomy during surgical treatment for congenital heart disease. The secondary objectives were to evaluate the deformity's type, flexibility, severity, and radiographic changes in these patients.

## Materials and Methods

The clinical data for assembling groups with and without sternotomy were collected from the medical records of patients with Down syndrome. All legal guardians were informed about the study and provided informed consent. The evaluation protocol was approved by the Institutional Ethics Committee (37229014.0.1001.5553).

**Fig. 1:**
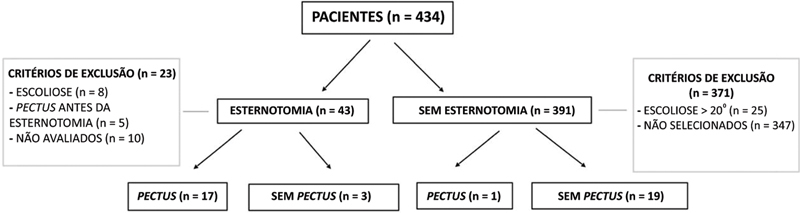
Flowchart of patient selection for the study groups (sternotomy and control groups).

The study included 434 medical records of Down syndrome patients, of which 43 had a history of sternotomy during childhood for cardiac repair. Fig. 1 shows the flowchart of patient selection for the study. There were 20 patients classified as the sternotomy group (8 males and 12 females; mean age 12.51, 1–24 years, standard deviation [SD]: 7.16) of whom 11 were children or growing adolescents and 9 were adults. Of the 391 remaining patients, 20 without a history of sternotomy were randomly selected as the control group (8 males and 12 females; mean age 14.67 years, 1–34 years, SD: 9.04), with 11 children or growing adolescents and 9 adults.

The mean age of sternotomy patients was 24.35 months. The patient who underwent sternotomy later was 96-months-old at the time of the sternotomy.

After the grouping of patients, clinical evaluations were initiated at the orthopedic outpatient clinic.

The patients' relatives were asked about the date of previous sternotomy, presence of pectus deformity prior to operation, time of pectus onset after , family history of chest deformities, and previous pectus treatment. The clinical evaluations were performed by a single pediatric orthopedist specialized in the noninvasive treatment of thoracic deformities. The analyses involved thoracic deformity type, severity (mild, moderate, or severe) and flexibility, sternal length (normal or short, with “short” being considered when the sternum or xiphoid process terminated proximal to the nipple line), and the presence of exacerbated thoracic kyphosis or scoliosis.

Deformities were classified as superior pectus carinatum (SPC), inferior pectus carinatum (IPC), lateral pectus carinatum (LPC), broad pectus excavatum (BPE), and localized pectus excavatum (LPE), as described in previous studies. 1 10 11 13

**Fig. 2:**
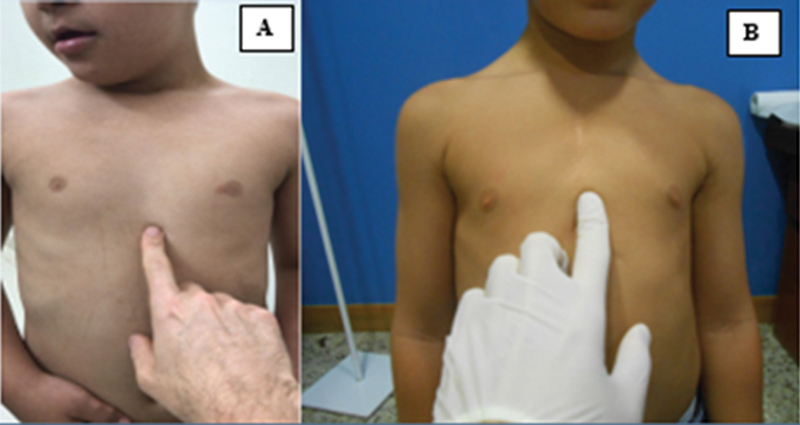
Evaluation of the sternal length in a patient with a sternal of normal length ( **A** ), and in a patient with shortened sternum ( **B** ).

The evaluation of the sternal length is shown in Fig. 2 . The pectus carinatum flexibility was evaluated by manual compression of the deformity at the apex in the anteroposterior direction. Pectus excavatum flexibility was evaluated by manual compression at the lower costal edge protrusions in the anteroposterior direction, with the patient simultaneously performing a Valsalva maneuver with arm adduction against resistance, while observing the effects on the depression area. 4 Patients who could not perform the Valsalva maneuver were instructed to either blow a balloon or exempt from this evaluation component.

For both pectus carinatum and excavatum, the deformity was classified as very or moderately flexible when there was a significant or complete reversal of the deformity, respectively. Alternatively, it was classified as less flexible when the pectus presented little change, or rigid when there was none with the provocative maneuvers performed,.

The chest imaging findings of all patients of the sternotomy and control groups were documented. Radiographs were taken in the lateral and oblique views of the sternum.

**Fig. 3:**
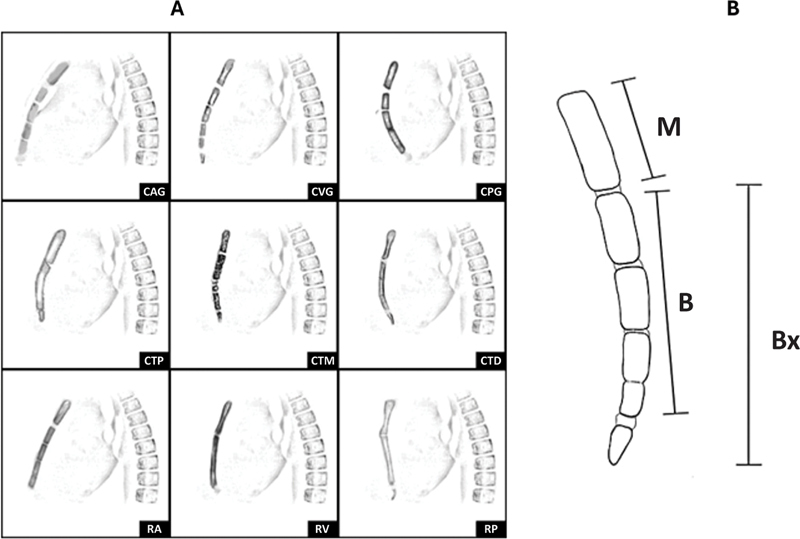
( **A** ) Illustration of the sternal patterns: gradual anterior curve (GAC), gradual vertical curve (GVC), gradual posterior curve (GPC), proximal third curve (PTC), middle third curve (MTC); distal third curve (DTC), anterior rectilinear (AR), vertical rectilinear (VR), and posterior rectilinear (PR) 14 . ( **B** ) Diagram of measurements taken from the lateral view of the sternum of a child with a normal chest, around 10-years-old, with two open growth plates. The length of the manubrium (M) and the body of the sternum (B), and the straight line drawn from the proximal end of the sternal body to the distal end of the xiphoid (Bx) were measured in centimeters. The result obtained by dividing the length of the sternal body (B) by the length of the manubrium (M) was called the BM index. When the xiphoid process was ossified, the BxM index was measured, represented by the division of Bx by M 5 .

The presence of latero-lateral irregularity of the sternal body was evaluated in the oblique view. 9 The radiographic parameters observed in the lateral view were the number of open sternal growth plates (0 to 3), total number of sutures in the growth plate, sternum sagittal angulation pattern, 14 and sternal body-manubrium (BM) or xiphoid-manubrium (BxM) indexes ( Fig. 3 ). 5 A previous study demonstrated that these two indexes were constant in normal patients, regardless of age, with values ranging from 2.16 ± 0.24 (BM) to 2.73 ± 0.31 (BxM). These normal ranges were applied in the present study. 5

Additional abnormal radiographic findings were noted. Although computed tomography (CT) chest scans were not requested, patients who underwent this examination for other reasons had their images analyzed.

Total spine radiographs in the orthostatic position were requested for patients with clinical examination findings suggestive of scoliosis or exacerbated kyphosis to determine the severity of the curve by the Cobb angle.

The Shapiro-Wilk test was used for the comparative statistical analysis between numerical variables of each group that followed normal distribution. When the hypothesis of normality was not rejected, the *t* test for independent samples was used to compare the groups. When the hypothesis of normality was rejected, the nonparametric Mann-Whitney test was used. The chi-squared test (x2) was used to compare categorical variables between groups.

The number of open sternal growth plates (0–3) and the presence of sutures variables were not compared for either group, as they were age-related and the number of children and adolescents in both groups was insufficient for a statistical analysis.

## Results

In the group of 20 patients who underwent sternotomy, 85% ( *n* = 17) presented with pectus deformities after the procedure. The mean time of onset was 5 months (median = 3 months), and 25% of the cases happened soon after ( *n* = 5); 15% ( *n* = 3) in 3 months; 5% ( *n* = 1) in 4 months; 5% ( *n* = 1) in 6 months; and 5% ( *n* = 1) in 36 months. In 30% ( *n* = 6) of the cases, family members failed to recall.

The mean age of pectus onset in our group was 27.47 months (2.29 years).

In the control group, the only patient with deformity presented with localized pectus excavatum at 6-months-old, with mild severity and moderate flexibility. None of the patients from either group had a family history of pectus deformities.

Table 1 shows the pectus types, severity, and flexibility in the sternotomy group.

**Table 1:** Pectus types, severity, and flexibility in the sternotomy group

Pectus type	Severity			Flexibility		
	Mild	Moderate	Severe	Moderate or high	Rigid or little	Total
IPC	11.8% (n = 2)	5.9% (n = 1)	11.8% (n = 2)	23.6% (n = 4)	5.9% (n = 1)	29.5% (n = 5)
LPC	29.5% (n = 5)	11.8% (n = 2)	0.0% (n = 0)	35.3% (n = 6)	5.9% (n = 1)	41.1% (n = 7)
SPC	5.9% (n = 1)	0.0% (n = 0)	0.0% (n = 0)	0.0% (n = 0)	5.9% (n = 1)	5.9% (n = 1)
LPE + LPC*	0.0% (n = 0)	5.9% (n = 1)	0.0% (n = 0)	0.0% (n = 0)	5.9% (n = 1)	5.9% (n = 1)
LPE	17.6% (n = 3)	0.0% (n = 0)	0.0% (n = 0)	11.8% (n = 2)	5.9% (n = 1)	17.6% (n = 3)
Total	64.8% (n = 11)	23.6% (n = 4)	11.8% (n = 2)	70.7% (n = 12)	29.5% (n = 5)	100.0% (n = 17)

**Abbreviations:** IPC, inferior pectus carinatum; LPE, localized pectus excavatum; LPC, lateral pectus carinatum; SPC, superior pectus carinatum. **Notes:** * Mixed type of pectus.

Mild scoliosis was found in 40 ( *n* = 8) and 20% ( *n* = 4) of patients from the sternotomy and the control groups, respectively, with statistical difference ( *p* < 0.001). All patients in both groups had postural kyphosis.

**Fig. 4:**
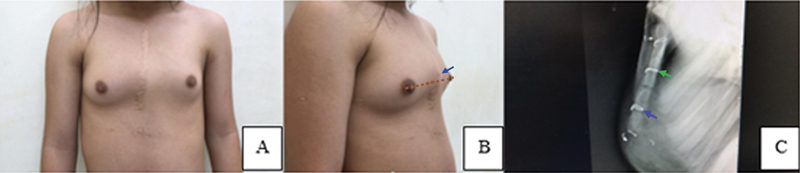
( **A** ) Anterior view of a 13-year-old patient who underwent sternotomy at 18 months of age. Right oblique view showing IPC. ( **B** ) The distal end of the sternum or xiphoid (apex of the deformity – arrow 1) was proximal to the nipple line (dashed line), showing clinical shortening of this bone. ( **C** ) Lateral view of the sternum showing the posterior angulation of the manubrium bone (arrow 1), metallic suture at the sterno-manubrial junction with incipient fusion (arrow 2), two open growth plates, metallic suture on the distal growth plate (arrow 3) with a rectilinear sternal pattern and BM index < 2.

All control patients had a clinically normal sternal length, while 40% ( *n* = 8) of the sternotomy group presented with a clinically shortened sternum; significant difference was observed between groups ( *p* = 0.01). Fig. 4 shows a patient in the sternotomy group with IPC and termination of the sternum at the level of the nipple, which represented sternal shortening ( Fig. 4A,B ), with a lateral radiographic view of the sternum showing several changes ( Fig. 4C ).

**Fig. 5:**
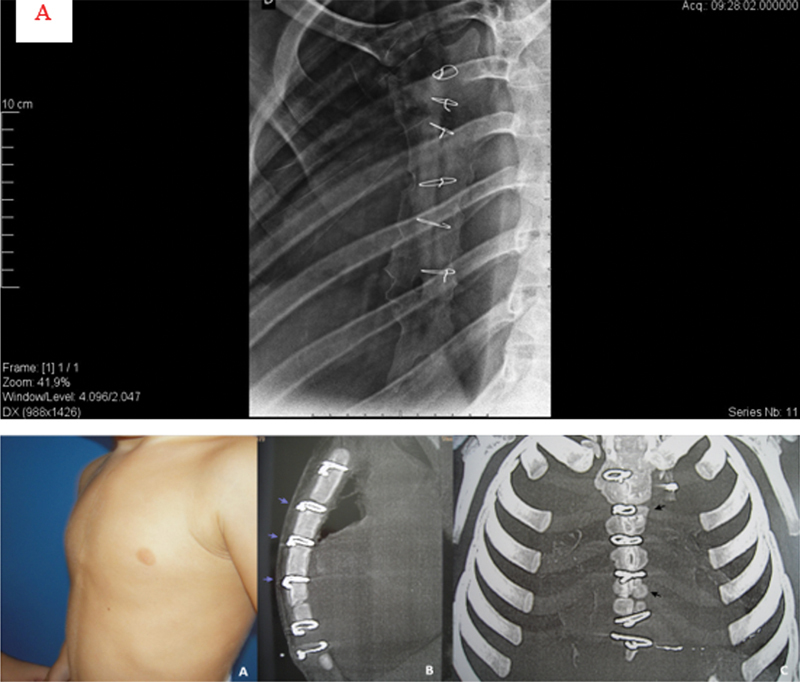
( **A** ) Oblique view of a 24-year-old patient with mild lateral pectus carinatum who underwent sternotomy at 96 months of age, with sterno-manubrial fusion and latero-lateral irregularities (asymmetries in the lateral contours on both sides of the sternum). ( **B** ) Computed tomography imaging of a two-year-old patient with IPC who underwent sternotomy ( **a** ). Sagittal image showing metallic structures in the two proximal growth plates and in the sterno-manubrial junction, with a GPC sternal pattern (arrows) ( **b** ). Coronal reformatted image showing irregular proximal growth plates and latero-lateral irregularity or asymmetry when comparing the two external sides (arrows) ( **c** ).

The measurement of the BM index showed normal sternal length in all patients without a history of sternotomy. In 38% ( *n* = 6) of the sternotomy group, the BM and BxM indexes showed a radiographically shortened sternum, which was significantly higher when compared with the control group (38 vs. 0%, *p* = 0.05). Oblique radiographic assessment of the sternum showed that latero-lateral irregularities were significantly higher in the sternotomy group (70 vs. 20%, *p* = 0.01), as shown in Fig. 5 .

As for the presence of metallic sutures in the growth plates, they were found in sternotomy patients who were still growing and with sternal growth plates still open ( *n* = 11). Of them, 36% ( *n* = 4) presented with sutures in the growth plates: two patients with sutures in two growth plates and two with sutures in one growth plate. One of these patients underwent CT in another hospital ( Fig. 5 ). All patients without pectus deformities in the sternotomy group ( *n* = 3) were already adults during radiographic evaluation, rendering it impossible to determine whether the metal sutures were previously placed on the referred growth plates. The BM and BxM indexes, the presence of latero-lateral irregularities, and the number of open sternal growth plates in the sternotomy and control groups are detailed in Table 2 .

**Table 2:** Results of the BM index

Radiograph findings		Group		
		Control	Sternotomy	Total
**BM/BxM indexes**	< 2.16 or < 2.73*	0% (n = 0/15)	38% (n = 6/16)** ^s^	20% ( *n* = 6/31)
	< 2.16 or < 2.73*	100% (n = 15/15)**	63% (n = 10/16)** ^s^	80% ( *n* = 25/31)
**Latero-lateral irregularity**	Yes	20% (n = 4/20)	70% (n = 12/17) ***	43% ( *n* = 16/37)
	No	80% (n = 16/20)	30% (n = 5/17) ***	57% ( *n* = 21/37)
**Growth plates open** ****	1	15% (n = 3/20)	0% (n = 0/20)	7.5% ( *n* = 3)
	2	10% (n = 2/20)	10,0% (n = 2)	10% ( *n* = 4)
	3	30% (n = 6/20)	20% (n = 4/20)	25% ( *n* = 10)
**Growth plates** **closed**	(n = 20)	45% (n = 9/20) adults 0% (n = 0/20) children ^&^	45% (n = 9/20) adults 25% (n = 5) children ^&^	57.5% ( *n* = 23)

**Abbreviations:** BM, body-manubrium; BxM, body xiphoid-manubrium. **Notes:** * < or > 2.16 values relative to the BM index and < or > 2.73 values relative to the BxM index 5 . ** 5 patients were excluded for presenting sterno-manubrial fusion causing inability to measure the BM or BxM indexes. ** ^s^ 4 patients were excluded for presenting sterno-manubrial fusion causing inability to measure the BM or BxM indexes. *** Failure of analysis in three sternotomy patients due to bad quality radiographs. **** All patients with growth plates open were children. ^&^ Patients with all growth plates closed, 0% ( *n* = 0/9) and 36% ( *n* = 5/14) were children from the control and sternotomy group, respectively.

In the sternotomy group, 2 patients (10%) presented with posterior angulation of the manubrium in the middle third segment (none in control group), one of whom associated with ossification in the anterior region, 20.0% ( *n* = 4) had sternomanubrial fusion (only two were children with pectus deformities, the others were adults, one without deformity), and 10.0% ( *n* = 2) presented with additional acute angulation of the sternum in the distal third portion, one of whom had anterior and posterior angulation. In the control group, 25% ( *n* = 5) presented with sternomanubrial fusion, all of them adults.

About the type of sternal curvature, the sternotomy group presented with 25% ( *n* = 5) of GAC, 5% ( *n* = 1) of posterior gradual, 30% ( *n* = 6) of GVC, 25% ( *n* = 5) of AR, 5% ( *n* = 1) of PR, and 10% ( *n* = 2) of vertical rectilinear curve. In the control group, 50% ( *n* = 10) had GVC, 40% ( *n* = 8) AR, and 10% ( *n* = 2) PR. The X2 test showed no significant difference regarding the type of sagittal angulations among and between the groups ( *p* = 0.20).

**Fig. 6:**
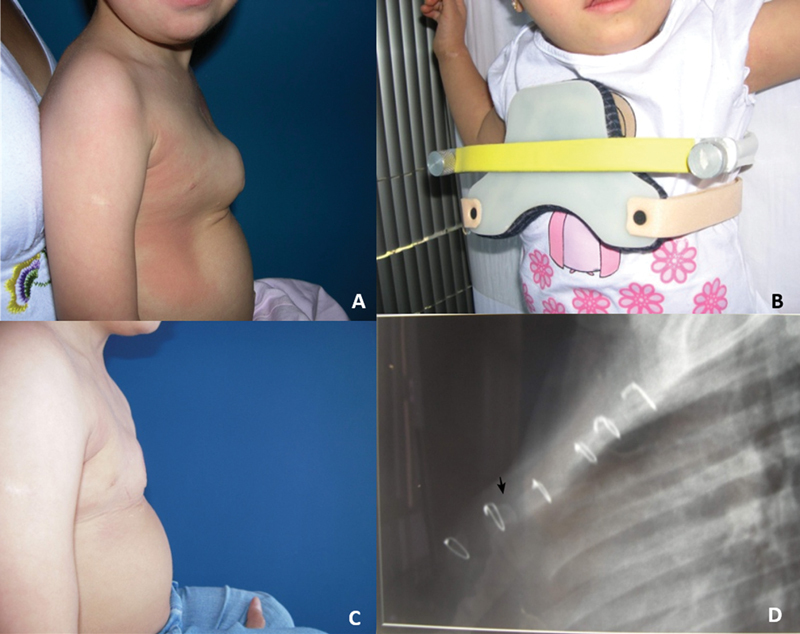
Patient with Down syndrome who underwent sternotomy for cardiac repair at 6 months of age and again at 13 months of age presented with ( **A** ) severe inferior pectus carinatum at 2 years and 1 month of age, ( **B** ) being initiated the use of a DCC 1 orthosis, ( **C** ) with good partial correction after a 24-month follow-up. ( **D** ) Lateral radiography of the sternum showing an AR pattern with additional acute anterior angulation in its distal third portion (arrow) and early sterno-manubrial fusion.

Of the sternotomy group patients who developed pectus deformities, three were treated using an orthosis ( Fig. 6 ).

## Discussion

This is the first study to analyze the prevalence of pectus deformity in patients who underwent sternotomy. Our finding of the high prevalence of such deformities in sternotomy patients (85%) propose that families need to be informed about this risk before the referral to cardiac surgery needing sternotomy.

In the present study, 25.0% ( *n* = 5) of the patients who underwent sternotomy were diagnosed with pectus deformity shortly after the procedure, inferring an immediate postsurgical change in the sternal bone. Additionally, the mean age of onset was 2.5 years in the patients' following sternotomy, which was much earlier than idiopathic cases which mostly appeared in the preadolescence period. 4 9

According to Ellis, 40% of patients had a family history of pectus deformity. 15 However, these differed from the sample analyzed in our study, in which none of the patients with pectus deformity had a family history, reinforcing the iatrogenic etiology.

Pectus excavatum is the most frequently reported type in prevalence studies, 16 although a study by Haje et al. 17 on 4,012 patients reported that 79% had pectus carinatum (IPC: 45%, LPC: 28%, SPC: 5%, LPE: 13%, and BPE: 9%). 17 The comparison between this study series of iatrogenic cases with those reported by Haje and Haje 17 showed that LPC was the predominant pectus type, which can be justified by an eventual irregular apposition between the two sternal sides when surgically closing the sternum.

The study by Haje et al. 5 showed no sutures between the bone segments of the sternum as previously described by Currarino and Silverman, 5 18 but the presence of growth plates. The sternum length increases due to their action. These plates are also responsible for the growth of costal cartilages and ribs in the anterior chest wall. Any surgical aggression to these structures can result in disproportionate growth and, consequently, deformities. 1 5 7 10 In the present study, 70.0% of sternotomy pectus patients ( *n* = 12) had latero-lateral irregularities, early growth plate closure, sternomanubrial fusion, and other changes that were not found in the control group, such as posterior angulation of the manubrium, acute angulation of the distal third of the sternum, and irregular ossification anterior to the sternal body.

A previous study had CT scans with coronal reconstruction showing that lateral sternal body irregularities were more frequent in pectus patients than in the control group ( *n* = 10). Furthermore, these irregularities were more difficult to interpret and perform in oblique radiographs of the sternum. However, due to radiation concerns, chest CT for diagnosis was not routinely recommended. 10

Sternomanubrial fusion occurred more frequently in adult patients but also occurred in two pediatric patients in the sternotomy group. Generally, this fusion does not occur in children, and is only found in10 to 30% of cases in adulthood, 12 although an incidence of 55% was found in our adult control patients.

The present study included the clinical evaluation of sternal lengths, with 40.0% ( *n* = 8) of sternotomy patients reporting a shortened sternum, unlike the control group, in which none reported such findings. This description of the clinical length of the sternum has not been previously reported in the literature and the authors believe it should be included in the physical examination of these patients.

The sternotomy group had more radiographically shortened sternums (abnormal BM index) than the control group, reinforcing the concept that surgical aggression to the sternal growth plates can shorten this bone, generating deformity, mostly mild severity. The BM (index was previously used to evaluate the radiographic shortening of the sternum, suggesting that a disproportion between the sternal and costal growths may result in a pectus deformity. 5

This study also evaluated the number of open or closed sternal growth plates, which are scheduled to close according to age group, also varying individually. In the sternotomy group, five children with pectus deformity had all plates closed, which did not occur in the control group.

As for the treatment of pectus deformities, the use of DCCs and surgical correction have been described. 1 4 7 8 9 11 According to Haje et al., 7 9 flexibility is the most important prognostic factor for conservative treatment. Idiopathic IPC and LPC are the most flexible types of carinatum, while SPC is more rigid and resistant to orthotic treatment. 9 17 However, two LPC and IPC cases in this study had rigidity during childhood, which was an unusual presentation. Therefore, iatrogenic pectus deformities tend to be more rigid than their idiopathic counterparts.

Aware that lesions in growth cartilage plates can cause deformity in the short and long term, orthopedic surgeons usually try to preserve them when operating on long bones in children and adolescents. Such care should be extended to surgeries performed on all bones in the thoracic region. Therefore, improved sternal suture techniques may decrease the onset of pectus deformity, saving the sternal growth plates or making their apposition as accurate as possible during sternal suturing.

## Conclusion

In conclusion, sternotomy in Down syndrome patients was associated with a high prevalence of pectus deformity, most of which were mild and of the LPC type, with radiographic changes.
